# Effectiveness of Pediatric Asthma Pathways in Community Hospitals: A Multisite Quality Improvement Study

**DOI:** 10.1097/pq9.0000000000000355

**Published:** 2020-10-26

**Authors:** Mansi Desai, Katherine Caldwell, Nisha Gupta, Arpi Bekmezian, Michael D. Cabana, Andrew D. Auerbach, Sunitha V. Kaiser

**Affiliations:** From the * Department of Pediatrics, University of California, San Francisco, California; † Department of Epidemiology and Biostatistics, University of California, San Francisco, California; ‡ Department of Medicine, University of California, San Francisco, California

## Abstract

Pathways guide clinicians through evidence-based care of specific conditions. Pathways have been demonstrated to improve pediatric asthma care, but mainly in studies at tertiary children’s hospitals. Our global aim was to enhance the quality of asthma care across multiple measures by implementing pathways in community hospitals.

**Methods:**

This quality improvement study included children ages 2–17 years with a primary diagnosis of asthma. Data were collected before and after pathway implementation (total 28 mo). Pathway implementation involved local champions, educational meetings, audit/feedback, and electronic health record integration. Emergency department (ED) measures included severity assessment at triage, timely systemic corticosteroid administration (within 60 mins), chest radiograph (CXR) utilization, hospital admission, and length of stay (LOS). Inpatient measures included screening for secondhand tobacco and referral to cessation resources, early administration of bronchodilator via metered-dose inhaler, antibiotic prescription, LOS, and 7-day readmission/ED revisit. Analyses were done using statistical process control.

**Results:**

We analyzed 881 ED visits and 138 hospitalizations from 2 community hospitals. Pathways were associated with increases in the proportion of children with timely systemic corticosteroid administration (Site 1: 32%–57%, Site 2: 62%–75%) and screening for secondhand tobacco (Site 1: 82%–100%, Site 2: 54%–89%); and decreases in CXR utilization (Site 1: 44%–29%), ED LOS (Site 1: 230–197 mins), and antibiotic prescription (Site 2: 23%–3%). There were no significant changes in other outcomes.

**Conclusions:**

Pathways improved pediatric asthma care quality in the ED and inpatient settings of community hospitals.

## Abstract 2

Supplemental Digital Content is available in the text.

## INTRODUCTION

Childhood asthma is a leading cause of emergency visits, hospitalizations, missed school days, and missed workdays for caregivers, with total estimated direct costs of approximately $6 billion annually in the United States.^1–3^ Evidence-based guidelines for asthma management are widely available.^4,5^ However, clinicians face many challenges adhering to guidelines,^6^ which contributes to poor health outcomes for the approximately 2 million children with asthma cared for in hospital and ED settings annually in the United States (eg, increased risk of unnecessary interventions, prolonged stay, hospital admission, transfer to intensive care, and hospital readmission).^7–9^

Pathways are a potential tool for improving evidence-based care and outcomes for children with asthma. *Pathways* are operational, bedside versions of evidence-based guidelines.^10^ They visually guide clinicians step-by-step through the timing, indications, and details of recommended tests and treatments for managing children with a specific illness. Pediatric asthma pathways commonly include instructions on assessing a child’s clinical severity and selecting and titrating therapies, such as bronchodilators (see **Appendix, Supplemental Digital Content 1,** http://links.lww.com/PQ9/A217). Pathways improve quality of care for children with asthma in the emergency department (ED) and inpatient settings (eg, increasing appropriate selection and timely administration of recommended medications; reducing the length of stay, costs, and healthcare disparities).^11,12^

To date, most studies of pediatric asthma pathways have been conducted in freestanding, tertiary children’s hospitals and children’s hospital-based EDs.^11,13–15^ However, less than 30% of children with asthma are cared for in these settings.^16^ It remains unclear if pathways’ effects on care and outcomes are generalizable to community hospital settings, which face unique challenges in pediatric quality improvement efforts, including more limited access to pediatric services and specialists, lower pediatric patient volumes, less quality improvement infrastructure, and higher prioritization of resources for adult care.^17,18^

In Fall 2017, 2 community hospitals that are part of an academically affiliated quality improvement (QI) consortium identified asthma care as a high priority area based on a high prevalence of asthma cases and a desire to standardize care across the ED and inpatient wards. The global aim of this quality improvement study was to improve the quality of asthma care across multiple measures by implementing pediatric asthma pathways in both the ED and inpatient wards of these community hospitals. SMART aims for the ED and inpatient setting are outlined in Table 1.

**Table 1.** Global and SMART Aims

Global Aim: To Improve the Quality of Hospital Care for Children with Asthma				
	BaselineSite #1	BaselineSite #2	Measure Type	Measure Calculation
SMART aims for EDs				
Increase assessment of asthma severity at triage by 10% within 12 mo	99%	100%	Process	Children with assessment of severity of asthma exacerbation at ED triage/All children
Increase the administration of systemic corticosteroids within 60 mins of triage by 20% within 12 months	28%	59%	Process	Children administered systemic corticosteroids within 60 mins/Children administered systemic corticosteroids
Decrease the utilization of CXR by 15% within 12 mo	48%	26%	Process	Children with CXR ordered during ED visit/All children
Decrease hospital admissions (including transfers for a higher level of care) by 3% within 12 mo	8%	15%	Outcome	Children with ED disposition of admission or transfer for a higher level of care/All children
No significant change to ED length of stay	226 mins	124 mins	Balancing	Mean length of stay (mins)
SMART aims for inpatient wards				
Increase the early transition to administering bronchodilator via metered-dose inhaler by 30% within 12 mo	61%	27%	Process	Children with first dose of MDI given at 1- or 2-h frequency or MDI ordered at hospital admission/ All children
Increase screening for secondhand smoke exposure by 10% within 12 mo	91%	52%	Process	Children with documented screening for secondhand tobacco smoke exposure/All children
Increase in documentation of caregiver referral to smoking cessation resources for eligible patients by 50% within 12 mo	0%	0%	Process	Children with caretakers referred to cessation resources/Children with caretakers that reported smoking
Decrease the prescription of antibiotics at discharge by 5% within 12 mo	7%	13%	Process	Children with any antibiotic prescribed at discharge/All children
Decrease the inpatient length of hospital stay by 10% within 12 mo	40 h	35 h	Outcome	Average length of stay (h)
No significant change to 7-d hospital readmission/ED revisits	5%	0%	Balancing	Children readmitted or seen in the ED for any indication within 7 d after hospital discharge/All children

Baseline values were determined using data from January to December 2017, and SMART aims were determined before calculation of baseline values. After baseline performance at each hospital was quantified, implementation teams focused PDSA cycles on higher priority measures.

## METHODS

### Context/Setting

Our study took place in 2 private, nonprofit, academically affiliated community hospitals. Hospital 1 is in an urban area with 685 total beds, 8 pediatric beds, and a pediatric intensive care unit. Hospital 2 is in a suburban area with 415 total beds, with ten inpatient pediatric beds and no pediatric intensive care unit. In both hospitals, children admitted to the inpatient setting are cared for by pediatric hospitalists. Children in the ED are cared for by emergency medicine physicians (<10% board-certified in pediatric emergency medicine at either site). Hospital 1 has daytime pediatric attending coverage and night coverage by an in-house pediatric resident remotely supported by a pediatric attending (via phone), and Hospital 2 has continuous pediatric attending coverage. Both sites also have medical trainees assisting in the care of children. Hospital 2 had a pediatric asthma electronic order set in place before this study, but Hospital 1 did not. Neither site had engaged in QI projects or implemented other interventions focused on improving care for children with asthma before this study.

### Study Design and Population

For this quality improvement study, we collected data through a chart review of ED visits and inpatient admissions. Chart review included children ages 2–17 years with a primary diagnosis of asthma. We excluded charts if children had chronic medical conditions that precluded pathway use such as chronic lung disease (eg, cystic fibrosis, restrictive lung disease, and bronchopulmonary dysplasia), congenital or acquired heart disease, airway issues (eg, vocal cord paralysis, tracheomalacia, and tracheostomy dependence), immune disorders, sickle cell anemia, or neuromuscular disorders. Each hospital reviewed all eligible inpatient admissions, and ED visits were reviewed up to a max of 20 per month. We defined baseline performance using data from January to December 2017 and collected data on the effects of pathway implementation from January 2018 to April 2019. The institutional review board of the University of California, San Francisco, approved the study. We report our study using the SQUIRE 2.0 guidelines.^19^

### Intervention

Study/QI project efforts began in Fall 2017. At each participating hospital, a physician implementation leader recruited a multidisciplinary team that included physicians, hospital administrators, nurses, pharmacists, and respiratory therapists to oversee pathway implementation. These teams adapted externally developed pediatric asthma pathways [from the American Academy of Pediatrics (AAP)]. The pathways guided clinicians through evidence-based practices in pediatric asthma care (see **Appendix, Supplemental Digital Content 1,** http://links.lww.com/PQ9/A217). Implementation teams led educational meetings to review pathway content and regularly conducted audit and feedback with physicians, nurses, and respiratory therapists. Audit and feedback involved review of run charts of hospital-specific performance on all quality measures.

**Fig. 1.**
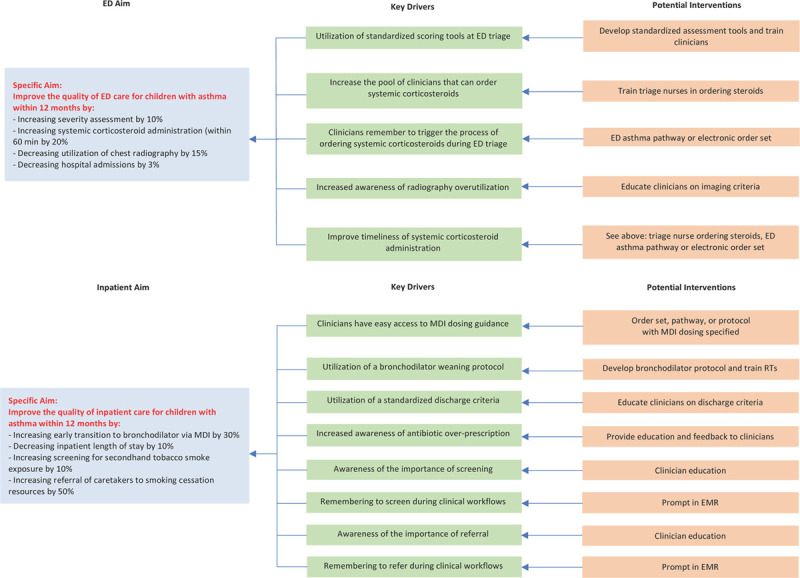
Key driver diagram. Blue boxes illustrate the study’s aims, green boxes illustrate key drivers, and orange boxes illustrate interventions. EMR, electronic medical record; RT, respiratory therapist.

**Fig. 2.**
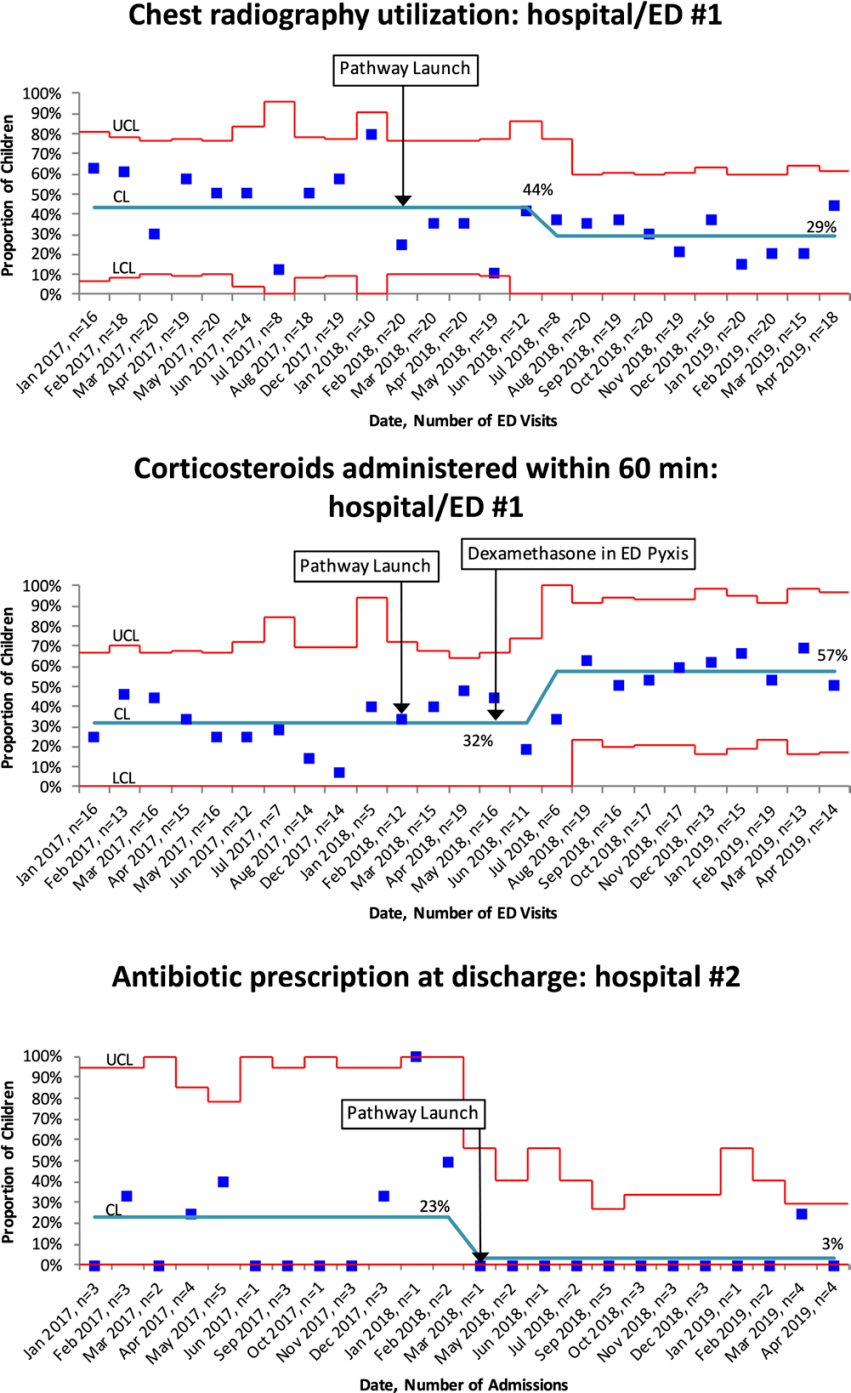
Effects of pediatric asthma pathways in community hospitals. Statistical process control charts of all study measures that showed significant changes in the ED and inpatient setting. Centerline (CL) represents mean performance over time. Red lines represent control limits, and individual data points represent mean performance from each month of the study. Centerline and control limits were shifted when the measure met criteria for special cause variation based on a run of eight or more data points in a row above/below the centerline.

After defining aims, implementation teams collected baseline data on the selected quality measures. After reviewing baseline performance on these measures, teams met monthly and used the Model for Improvement and key driver diagram (Fig. 1) to design Plan-Do-Study-Act (PDSA) cycles.^20^ PDSA cycles for each hospital are detailed in **Table 2 (Supplemental Digital Content 1,** http://links.lww.com/PQ9/A217) and Figures 2 **and 3 (Supplemental Digital Content 1,** http://links.lww.com/PQ9/A217). Improvement cycles began in February 2018 and concluded in December 2018. The effects were monitored until April 2019.

Interventions at Hospital 1 included education/training on pathways and posting of pathways in work areas for visual reference (both the ED and inpatient setting). Additionally, in the inpatient setting, teams modified the electronic health record (order sets and note templates). Interventions at Hospital 2 included education/training on pathways, posting pathways in work areas for visual reference, and modification of the electronic order sets and nursing flowsheets (both the ED and inpatient setting). Additionally, in the inpatient setting, there was the initiation of a respiratory therapy-led bronchodilator weaning protocol. In the ED, there was the addition of a floating ED attending to assess children at triage. At each hospital, implementation teams discussed and selected these interventions based on local quality measure performance and context (eg, workflows and resources). For example, in both EDs, interventions for pathway launch (educational sessions and posting of pathways in workspaces) did not change rates of timely systemic corticosteroid administration. Consequently, implementation teams discussed workflow barriers and resources at each site. ED #1 found dexamethasone delivery from the pharmacy was a limiting factor, so the next intervention pursued was stocking dexamethasone in the ED triage automated medication dispensing station. ED #2 found physician assessment was a limiting factor and was able to add a floating ED attending that helped expedite this task.

Ongoing plans for sustainability included electronic health record integration (order sets, flowsheets, and note templates) and local champions [physicians, nurses, and respiratory therapists that continue to remind clinicians of pathway recommendations and educate and train new staff (volunteer commitment of approximately 2 h/mo)].

### Measures/Outcomes

In Fall 2017, implementation teams selected measures using external resources from the AAP. The global aim was to improve the quality of asthma care. SMART aims were selected before the chart review and analysis of baseline performance (Table 1). After baseline performance at each hospital was quantified, implementation teams focused PDSA cycles on higher priority measures. For the ED, we focused on administering systemic corticosteroids within 60 minutes of arrival (process measure). This metric has been associated with reductions in time to recovery and risk of hospital admission.^21^ Other measures included severity assessment at ED triage (process measure), utilization of chest radiography (CXR) (process measure), and hospital admission (outcome measure). We examined ED length of stay (LOS) as a balancing measure to ensure it did not increase with the implementation of new clinical workflows. For the inpatient setting, we focused on early administration of metered-dose inhalers (MDIs) as a process measure because MDIs are more cost-effective and have fewer side effects than nebulizers,^4^ and MDI use promotes asthma self-management education and better asthma control.^22^ Other measures included screening for secondhand smoke exposure (process measure), referral of caregivers to smoking cessation resources (process measure), antibiotic prescription at discharge (process measure), length of hospital stay (outcome measure), and 7-day hospital readmissions or ED revisits (balancing measure).

### Analysis

We compared demographic and clinical characteristics between the prepathway and postpathway groups using Student’s *t*-tests and χ^2^ tests (or Fisher’s exact tests for comparisons with n < 5). Statistical significance was set at α = 0.05. We used statistical process control charts to determine the absolute changes in outcomes associated with pathway implementation. We used X-bar S charts to analyze the length of ED and inpatient stay (continuous data) and P charts to analyze all other outcomes (count data on both conforming and nonconforming units).^23^ Control limits were set at 3 standard deviations from the mean. Standard rules were used to determine special cause variation (eg, ≥8 points above/below the baseline centerline, six consecutive points increasing or decreasing).^23^ Centerlines and control limits were adjusted when a shift was detected (>8 points above/below the baseline centerline).

## RESULTS

### Study Population

Within the 2 community hospitals, there were 881 ED visits and 138 hospital admissions for children with a primary diagnosis of asthma during the study period (Table 2). The demographics of the overall study population reflect expected patterns in young children with asthma,^24^ with more males than females. Also, we found the proportion of males cared for in the ED was higher in the postintervention period compared with the preintervention period. However, the groups were otherwise similar in terms of age, insurance type, the severity of asthma exacerbation, and prior prescription of inhaled corticosteroids (a marker of chronic asthma severity).

**Table 2.** Patient Characteristics in Preintervention versus Postintervention Period

Children with ED Visits for Asthma Preintervention versus Postintervention (n = 881)			
Patient Characteristics	Preintervention (n = 422)	Postintervention (n = 459)	*P*
Male (%)*	248 (59%)	311 (68%)	**<0.01**
Mean Age (y, SD)†	7.9 (3.9)	7.7 (4.3)	0.49
Prior inhaled corticosteroid use (%)*	157 (37%)	172 (37%)	0.93
Insurance (%)*			0.33
Private	79 (19%)	78 (17%)	
Public	319 (76%)	360 (78%)	
Tri-care	3 (1%)	0 (0%)	
Other, self-pay, or unknown	20 (5%)	21 (5%)	
Asthma exacerbation severity (%)*			0.07
Mild	190 (45%)	188 (41%)	
Moderate	171 (41%)	223 (49%)	
Severe	57 (14%)	45 (10%)	
Not documented	4 (1%)	3 (1%)	
Characteristics of children with inpatient admissions for asthma preintervention versus Postintervention (n = 138)			
Patient Characteristics	Preintervention (n = 70)	Postintervention (n = 68)	*P*
Male (%)*	39 (56%)	43 (63%)	0.37
Mean age (y, SD)†	6.7 (3.9)	6.5 (4.3)	0.77
Prior inhaled corticosteroid use (n%)*	37 (53%)	40 (59%)	0.48
Insurance (%)*			
Private	23 (33%)	22 (32%)	1.00
Public	45 (64%)	44 (64%)	
Tri-care	1 (1%)	0 (0%)	
Other, self-pay, or unknown	1 (1%)	2 (3%)	

The preintervention period was from January to December 2017 and postintervention period was from January 2018 to April 2019.

Bold value indicates statistically significant.

*Analyzed using Chi-squared tests or Fischer’s exact.

†Analyzed using Student’s t-test.

### ED Outcomes

Annotated statistical process control charts of ED results are presented in Figures 2 and **3 (Supplemental Digital Content 1,** http://links.lww.com/PQ9/A217). Pathway implementation was associated with absolute increases in the proportion of children administered systemic corticosteroids within 60 minutes of arrival (Site #1: 32%–57%, Site 2: 62%–75%) and decreases in the proportion of children receiving chest radiographs (Site #1: 44%–29%) and ED LOS (Site #1: mean 230–197 mins). There was also a small increase in the assessment of severity at ED triage (Site #1: 98.6%–100%). Shifts in these outcomes were detected 2–6 months after pathway launch. We found no significant changes in hospital admission (outcome measure).

### Inpatient Outcomes

Annotated statistical process control charts of inpatient results are presented in **Figures 2 and 3 (supplemental** **http://links.lww.com/PQ9/A217**). Pathway implementation was associated with absolute increases in the proportion of visits screened for secondhand tobacco smoke exposure (Site #1: 82%–100%, Site #2: 54%–89%) and a decrease antibiotic prescription at discharge (Site #2: 23%–3%). Shifts in these outcomes were detected 0–2 months after pathway launch. We found no significant changes in the early administration of bronchodilator via MDI (process measure), referral of caregivers to smoking cessation resources (process measure), inpatient LOS (outcome measure), or 7-day hospital readmissions or ED revisits (balancing measure).

## DISCUSSION

In this multisite study of both the EDs and inpatient wards of community hospitals, we found pathways were effective in improving multiple measures of quality of care for children with asthma. In EDs with few pediatric emergency-trained physicians, we saw increases in the timely administration of systemic corticosteroids, and reductions in the use of CXR and length of ED stay. In inpatient wards, we found improvements in screening for secondhand tobacco exposure and decreases in antibiotic prescription at discharge. Our findings suggest that pathways’ effects may be generalizable to the ED and inpatient settings of community hospitals.

The few prior studies evaluating the effectiveness of pediatric asthma pathways in community hospitals align with our findings. A previous single-center study of pathway implementation in a community ED found similar improvements in timely steroid administration and found decreased hospital admission rates.^15^ Our lack of improvement in the hospital admission rate may be related to differential achievement in timely steroid administration; however, we cannot directly compare performance, as the measures selected for that study were different than in our study (mean time to administration in minutes versus the proportion of children administered systemic corticosteroids within 60 mins, respectively). In line with our findings, a prior study of inpatient asthma pathway implementation at 1 children’s hospital and 7 community hospitals within the Intermountain Health System found improvements in a composite measure of guideline adherence.^25^ This multicenter study,^25^ as well as another single-center study in a community hospital,^14^ found pathways were associated with decreases in LOS and the associated hospital costs. Our hospitals may have had less opportunity for improvement in LOS. These prior studies demonstrated reductions in length of stay from 49 to 45 hours^25^ and 45 to 35 hours,^14^ but the baseline lengths of stay in our study sites were only 40 and 35 hours. Our sample size may have limited our ability to detect changes in hospital readmission rates, a problem that is well recognized for community hospitals.^26^

We found differing effects in the two participating community hospitals. At Hospital/ED #1, we found significant improvements in severity assessment at ED triage, timely systemic corticosteroid administration, CXR utilization, ED length of stay, and secondhand tobacco screening. At Hospital/ED #2, we found significant improvements in timely corticosteroid administration, secondhand tobacco screening, and antibiotic prescription at discharge. Differences in baseline performance may have driven these differences in improvements. For instance, CXR utilization did not significantly decrease at Hospital/ED #2, but baseline utilization was much lower at baseline (48% at Hospital/ED #1 versus 26% at Hospital/ED #2). This baseline of 26% is already close to previously defined benchmarks of care quality (25% benchmark defined by Parikh et al).^27^ Similarly, antibiotic prescription rates at discharge were lower at baseline in Hospital #1 at 7%, versus 13% in Hospital #2. This lower baseline rate may have led to a ceiling effect, leaving little room for significant improvement.

Additionally, differences in improvements may have been driven by differences in the barriers faced at each site. For instance, implementation teams at Hospital/ED #1 encountered delays in efforts to modify the electronic health record and hurdles with changing nurses’ and/or respiratory therapists’ workflows around the use of MDI (because they perceived MDI administration to require more time than nebulized bronchodilators and to be more disruptive to patients during sleep). Implementation teams at Hospital/ED #2 dealt with complex institutional approval processes for changing standard workflows and faced barriers to engaging ED clinicians.

We found pathway implementation was associated with a 15% decrease in CXR utilization in Hospital/ED #1 (absolute reduction from 44% to 29%). Routine CXR is not recommended for children with asthma^5^ because they rarely have concomitant bacterial pneumonia^28^; inconclusive radiographs may also potentially lead to unnecessary antibiotic therapy. As part of pathway implementation, educational meetings were conducted in which clinicians were provided criteria sets for when to order CXR. Criteria included the 4 F’s (fever, focal examination findings, concern for foreign body, and failure to improve). Bekmezian et al^29^ found such criteria sets decreased unnecessary utilization of CXR, which equated to reduced exposure to ionizing radiation in childhood and decreased healthcare costs. Hospitals should consider the use of such criteria sets in future implementation efforts. These criteria sets were reviewed at both hospitals as part of educational efforts at pathway launch and included in visual banners with pathway content.

We found pathway implementation was associated with an increase in the timely administration of systemic corticosteroids in the ED (Site 1: 32%–57%, Site 2: 62%–75%). More timely corticosteroid administration has been associated with decreased time to recovery and risk of hospital admission.^21,30^ Prior studies that demonstrated timelier corticosteroid administration describe shifting the responsibility of ordering corticosteroids from physicians to triage nurses and moving the medication supplies to the ED triage area to promote more efficient retrieval.^15,21^ Neither of our study sites shifted corticosteroid ordering responsibility to nurses. However, one ED created a role for a floating ED attending that quickly assessed children in triage, which has not been described before. Another ED added corticosteroids to the triage automated medication dispensing station for quicker access. EDs might consider these workflow modifications to achieve improvements in the timely administration of recommended medications for children with asthma, and possibly other patient populations that require highly time-sensitive medications.

Our study is the first to evaluate the effects of pathways using a multicenter design, including ED and inpatient community hospital settings. However, our study has several limitations. First, given that both study sites were academically affiliated, our results may have limited generalizability outside of this setting. However, these community hospitals represented varied sizes, locations, and levels of pediatric infrastructure. Second, although we evaluated for several critical potential differences in the patient mix before and after pathway implementation, we were not able to account for other factors such as race, ethnicity, and co-morbid medical conditions. While statistical process control charts facilitate the rigorous evaluation of performance changes, they do not correct for secular trends (long-term market changes) in these outcomes. Thus, such trends may have also played a role in driving the improvements we report. Last, our study involved monitoring intervention effects for 16 months, and longer-term sustainability was not assessed. Critical areas for future work include: (1) multicenter studies of pediatric asthma pathway implementation across larger, more diverse groups of community hospitals; (2) utilization of methodology that better accounts for secular trends, such as randomized controlled trials or interrupted time-series studies; and (3) use of mixed methods (quantitative and qualitative) to identify implementation practices associated with successful pathway implementation and improvements in quality of care.

## CONCLUSIONS

Our study demonstrates that pediatric asthma pathways are effective in improving the quality of asthma care of children cared for in both the ED and inpatient settings of community hospitals. We provide details on both effectiveness and implementation to help administrators and clinicians seeking to improve care for children with asthma in community hospitals.

## DISCLOSURE

The authors have no financial interest to declare in relation to the content of this article.

## Supplementary Material


