# Supplementary figures and images for: Effectiveness of Pediatric Asthma Pathways in Community Hospitals: A Multisite Quality Improvement Study
Source: Pediatr Qual Saf. 2020 Oct 26;5(6):e355. doi: 10.1097/pq9.0000000000000355 (PMC7591126; doi:10.1097/pq9.0000000000000355)

## Pathway for Hospital #1

### PEDIATRIC ASTHMA INPATIENT PATHWAY

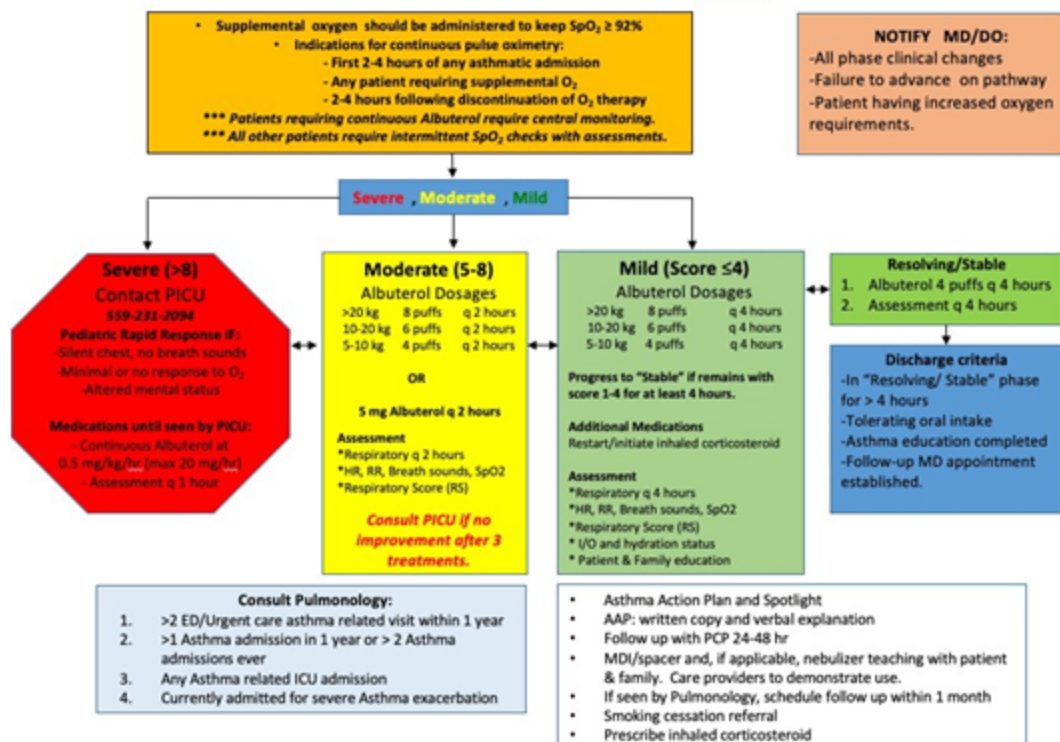

## Pathway for Hospital #2

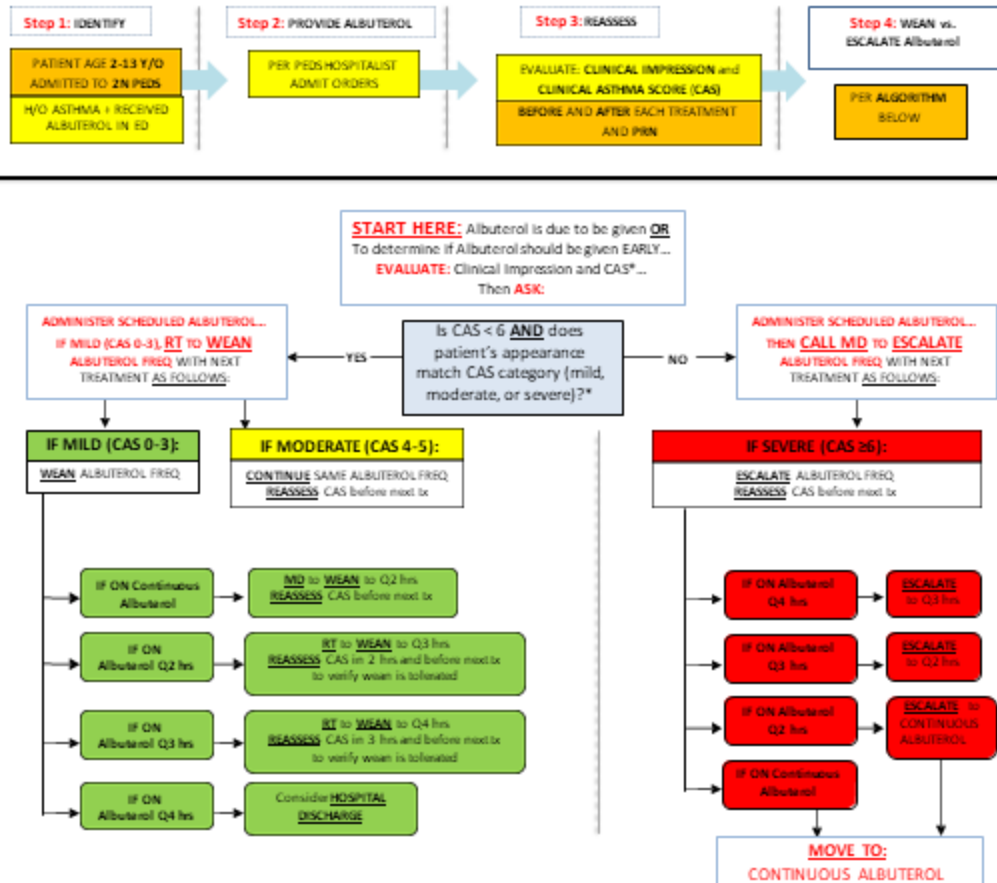

Supplement: Supplementary file 1 [file pqs-5-e355-s001.pdf]
